# Evidence for preferential copackaging of Moloney murine leukemia virus genomic RNAs transcribed in the same chromosomal site

**DOI:** 10.1186/1742-4690-2-3

**Published:** 2005-01-18

**Authors:** Sergey A Kharytonchyk, Alla I Kireyeva, Anna B Osipovich, Igor K Fomin

**Affiliations:** 1 Laboratory of Cellular and Molecular Immunology, Institute of Hematology and Blood Transfusion, 223059 Minsk, Republic of Belarus; 2 Present address: Department of Microbiology and Immunology, Vanderbilt University School of Medicine, Nashville, TN37232, USA

## Abstract

**Background:**

Retroviruses have a diploid genome and recombine at high frequency. Recombinant proviruses can be generated when two genetically different RNA genomes are packaged into the same retroviral particle. It was shown in several studies that recombinant proviruses could be generated in each round of HIV-1 replication, whereas the recombination rates of SNV and Mo-MuLV are 5 to 10-fold lower. The reason for these differences is not clear. One possibility is that these retroviruses may differ in their ability to copackage genomic RNAs produced at different chromosomal loci.

**Results:**

To investigate whether there is a difference in the efficiency of heterodimer formation when two proviruses have the same or different chromosomal locations, we introduced two different Mo-MuLV-based retroviral vectors into the packaging cell line using either the cotransfection or sequential transfection procedure. The comparative study has shown that the frequency of recombination increased about four-fold when the cotransfection procedure was used. This difference was not associated with possible recombination of retroviral vectors during or after cotransfection and the ratios of retroviral virion RNAs were the same for two variants of transfection.

**Conclusions:**

The results of this study indicate that a mechanism exists to enable the preferential copackaging of Mo-MuLV genomic RNA molecules that are transcribed on the same DNA template. The properties of Mo-MuLV genomic RNAs transport, processing or dimerization might be responsible for this preference. The data presented in this report can be useful when designing methods to study different aspects of replication and recombination of a diploid retroviral genome.

## Background

Retroviruses are a family of RNA viruses which replicate through a DNA intermediate [1]. The unique property of retroviruses is that their virions contain two identical genomic RNA molecules noncovalently linked near the 5' ends forming a dimer [2,3]. Thus, the retroviral genome is diploid. The presence of two RNA molecules in each virion seems to be necessary for recombination because there is no pool of viral replicative intermediates in the cells infected by retroviruses [4,5]. Recombination is thought to contribute to the genetic variability of retroviruses and to repair breaks in genomic RNA. It can not be excluded that both RNA molecules are necessary for synthesis of proviral DNA.

Reverse transcription entails two DNA strand transfers during minus and plus DNA synthesis. Since the retroviral virion contains two molecules of the viral RNA, the first DNA transfer might be either intramolecular, transferring to the same template, or intermolecular, transferring to the other template. In the model of Spleen necrosis virus (SNV) it was found that the minus-strand DNA transfer is exclusively intermolecular [6], while another study demonstrated the almost complete preference for intramolecular minus-strand transfer [7]. However, recombinant proviruses can undergo both interstrand and intrastrand transfers in equal proportions [7-9]. The rate of recombination in these reports was 4% per kilobase per replication cycle [4,8] and it was not significantly increased when the marker distance was extended to the size of the retroviral genome, suggesting that recombination is limited to only a subpopulation of retroviruses [10]. On the other hand, Human immunodeficiency virus type 1 (HIV-1) was shown to undergo approximately two to three recombination events per genome per cycle of replication [11] and, similar to the recombinant SNV proviruses, the first DNA strand transfer was either intra- or intermolecular [12,13].

A reason why there are differences in the rates of recombination between HIV-1 and gammaretroviruses (SNV and Mo-MuLV) is not known. It has been suggested that these differences may be associated with the differences in the template switching frequencies of retroviral reverse transcriptases [11]. A recent study has shown that the rates of intramolecular template switching for HIV-1 and Mo-MuLV (Moloney murine leukemia virus) were very similar, indicating that the replication properties of HIV-1 and Mo-MuLV RTs may not differ [14]. However, it is not clear whether the same conditions are required when both genomic RNAs are used as the template during reverse transcription. The other possibility is that gammaretroviruses may copackage genomic RNAs produced at different chromosomal loci by nonrandom chance [15]. In this case, the sizes of heterodiploid and recombining subpopulations of viruses may coincide.

In this study, we have investigated whether there is a preference in the formation of homodiploid virions during the mixed retroviral infection. To explore this possibility, we have used the forced recombination system which included two Mo-MuLV-based retroviral vectors containing different selectable markers and one of the vectors having a deletion of the PBS region. These vectors were introduced into the packaging cell line using two different methods, cotransfection, to provide tandem integration, or sequential transfection, and the frequencies of recombination for the vectors have been compared.

## Results

### Experimental approach

To study whether there was a preference for the formation of homodiploid virions in the mixed retroviral infection we have used two different methods, cotransfection and sequential transfection, to introduce genetically different retroviral vectors into the host cells. Since plasmid DNA transfected into eucariotic cells is usually tandemly integrated in a chromosome [16-19], it is expected that cotransfected vectors will be localized in the same locus of chromosome and RNA transcribed from these templates will form a general pool of molecules. In this case, two genetically different populations of RNA molecules will ideally overlap. On the other hand, it is unknown whether the same conditions exist for reassortment of RNA molecules transcribed at different chromosomal locations. The study of recombination frequencies for retroviral vectors that are introduced by the cotransfection or sequential transfection can help to answer this question.

### Comparative study of recombination frequencies for retroviral vectors with the same and different chromosomal locations

**Figure 1:**
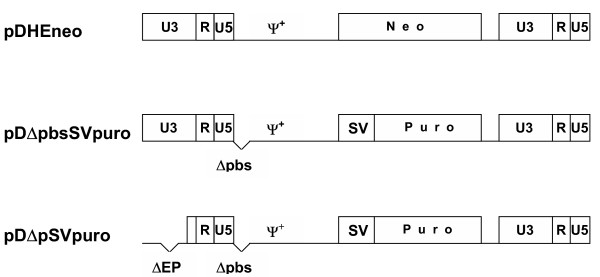
Structures of Mo-MuLV-based retroviral vectors used in this study. U3, R, U5, regions of long terminal repeat; SV, simian virus 40 early promoter region; ψ+, extended packaging signal; Neo, neomycin phosphotransferase gene; Puro, puromycin N-acetyltransferase gene. Δpbs and ΔEP indicate that the entire primer binding site and enhancer-promoter sequences from the U3 region are deleted.

In this study Mo-MuLV-based retroviral vectors were used as partners for recombination. These vectors contained the Mo-MuLV sequences as follows: the 5' and 3' LTRs, ψ region, a part of gag-sequences before XhoI site (position 1560 [20]), and 140 bp including the polypurine tract before 3' LTR (Figure 1). To selectively introduce vectors into the packaging cell line, pDHEneo contained the neo gene that was expressed by transcripts initiated from the long terminal repeat, while pDΔpbsSVpuro contained the puro gene under control of SV40 early promoter region. In addition to the differences in selectable markers, the pDΔpbsSVpuro vector was replication defective due to the deletion of entire PBS.

Since pDΔpbsSVpuro RNA is impaired at the initiation of reverse transcription, this function can be restored when the cDNA initiated on the copackaged pDHEneo RNA is transferred to the puro RNA template during the first jump; minus-strand synthesis continues through the puro gene, and the template shift occurs within the leader region. Thus, the restoration of retroviral vector containing the puro gene is possible via homologous recombination with the neo-containing construct at the sequence identity in the leader region of the genome.

**Figure 2:**
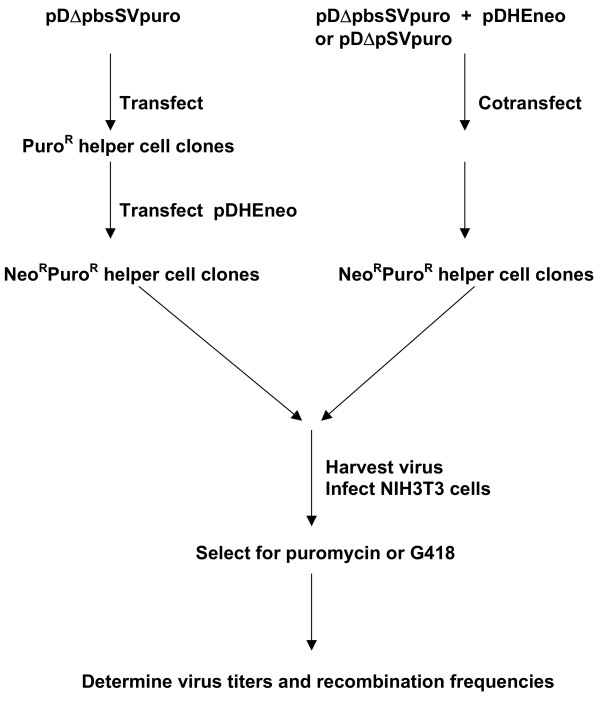
Experimental scheme to study recombination frequencies for retroviral vectors located in the same or different chromosomal sites.

The experimental scheme employed in this study is outlined in Figure 2. Retroviral vectors pDΔpbsSVpuro and pDHEneo were introduced into GP+envAM12 packaging cells by either the cotransfection or sequential transfection procedure. For sequential transfection pDΔpbsSVpuro was first introduced into helper cells. The transfected cells were placed on puromycin selection and the resistant cell clones were picked. Viral titers generated from these clones were analyzed using NIH3T3 cells. None of the cell clones analyzed produced detectable level of puromycin titer. Two clones were further used for transfection of pDHEneo and the G418 resistant clones were selected. For cotransfection the equal quantities of vector DNA was used for transfection of helper cells. The cells were first placed on G418 selection and the resistant cell clones were further obtained via puromycin selection. After drug selection, the double-resistant helper cell clones were isolated.

It was expected that plasmid DNA of retroviral vectors pDHEneo and pDΔpbsSVpuro cotransfected into the packaging cell line would be tandemly integrated into the host genome. To study the integration of plasmid DNA, the PCR analysis was performed with the primers hybridizing to the 3' end of neo gene (T1, direct, for pDHEneo) and to the SV40 early promoter region (T2, reverse, for pDΔpbsSVpuro). Using these primers, the specific PCR products could be obtained if the pDHEneo and pDΔpbsSVpuro are located in the same chromosomal site. On the other hand, PCR products could be generated with only one of the primers when identical molecules of plasmid DNA were integrated in the opposite orientation. However, the efficiency of amplification in this case seems to be very low because such sequences will contain inverted repeats.

**Figure 3:**
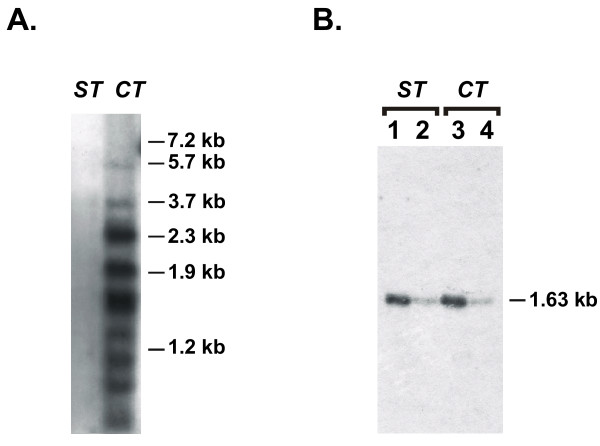
PCR analysis of plasmid DNA transfected into the packaging cell line GPenv-AM12. **A. **Analysis of tandemly integrated plasmid DNA. Amplification was performed with a 5' primer specific to neo sequences (T1, unique for pDHEneo) and a 3' primer specific to SV40 early promoter region (T2, unique for pDΔpbsSVpuro). Membrane was hybridized with 3' neo specific probe generated from a 150 bp SalI-ClaI fragment of pDHEneo. **ST **is GPenv-AM12 virus-producing cell clone ST2-1 generated by sequential transfection of pDHEneo and pDΔpbsSVpuro, and **CT **is cell clone CT2 generated by cotransfection of the same vectors. Molecular size markers are indicated on the right of the Southern blot. Similar results were obtained when four cell clones were analyzed. **B. **Control of amplification. Primers specific to the 5'- and 3'-end of neo gene (CND and CNR, respectively) were used to generate PCR products (1.63 kb) from ST and CT DNA samples. Membrane was hybridized with the same probe as in **A**. PCR products obtained from 200 and 40 ng of ST DNA sample (line 1 and 2); PCR products obtained from 200 and 40 ng of CT DNA sample (line 3 and 4). The result shows that specific PCR products could be amplified both from ST and CT DNA samples with this set of primers.

The PCR analysis was performed using chromosomal DNA prepared from different cell clones generated after cotransfection or sequential transfection of vectors. PCR products were separated by gel electrophoresis, transferred onto nylon membrane and hybridized with 3' neo specific probe. An example is presented in Figure 3 which shows that specific PCR products of different size were obtained only for the cell clone generated after cotransfection of two vectors. These data are in agreement with early observations [16-19] and demonstrate that plasmid DNA transfected into the packaging cells is cointegrated into the cellular DNA.

**Figure 4:**
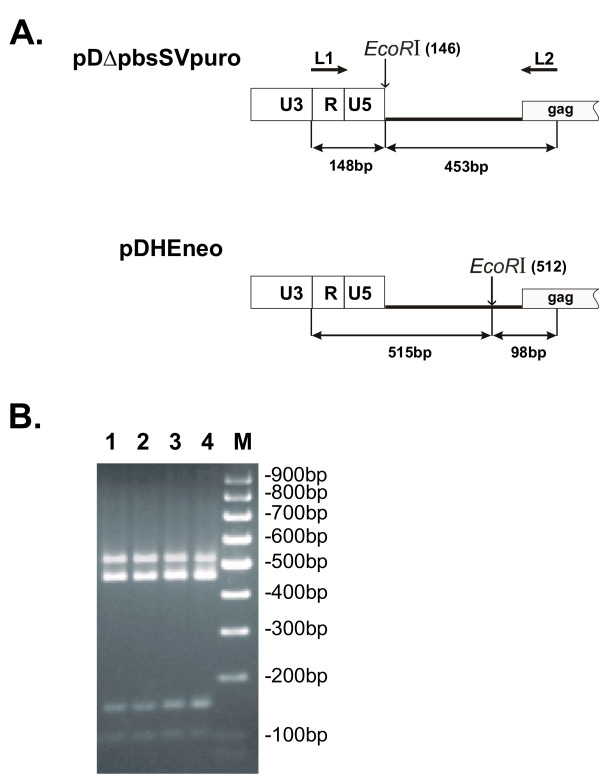
RT-PCR analysis of virion RNAs. **A. **Plasmid structures of retroviral leader regions. L1 and L2, primers used for PCR amplification; sizes of DNA fragments and positions of EcoRI sites are indicated. **B. **Leader sequences in virion RNAs were PCR amplified and analyzed by restriction digestion. PCR products obtained from virion RNAs of ST2-1 and ST2-2 packaging cell clones (lines 1 and 3); PCR products obtained from virion RNAs of CT1 and CT2 cell clones (lines 2 and 4); M, molecular weight markers. The ratios of puro/neo retroviral RNAs for ST2-1, ST2-2, CT1, and CT2 cell clones were 1.8, 2.0, 1.6, and 2.5, respectively.

We also used RT-PCR-based assay to examine the ratios of retroviral virion RNA molecules for cell clones generated by different methods of transfection. Since retroviral vectors differed by localization of EcoRI sites in the leader regions, these restriction sites were used as markers to distinguish the two coamplified PCR products obtained with primers specific to this region (Figure 4). EcoRI digestion generated 453- and 148-bp fragments from the pDΔpbsSVpuro PCR products that were readily distinguishable from the 515- and 98-bp fragments generated from the pDHEneo PCR products. Since the only differences between the neo- and puro-containing RNAs are nineteen bases that lie within the polymerized region (PBS was replaced with EcoRI in pDΔpbsSVpuro and one nucleotide was substituted in the leader region of pDHEneo to introduce EcoRI site), these two templates will amplify with equal efficiency. PCR products obtained from virion RNA for the two cell clones generated by sequential transfection and two clones generated by cotransfection of retrovital vectors were digested with EcoRI and the ratio of corresponding DNA fragments was examined. This analysis showed that ratios of retroviral RNAs for different cell clones ranged from 1.6 to 2.5 (pDΔpbsSVpuro/pDHEneo) and were the same for two variants of transfection (Figure 4).

Viral titers generated from three helper cell clones obtained after sequential transfection and four cell clones obtained after cotransfection are shown in Table 1. In the first case the G418 titers varied from 5.0 × 10^3 ^to 6.3 × 10^4 ^CFU/ml and puromycin titers from 5.1 × 10^1 ^to 8.0 × 10^2 ^CFU/ml. In the cotransfection experiment, the G418 titers varied from 3.1 × 10^4 ^to 1.1 × 10^5 ^CFU/ml and puromycin titers from 1.4 × 10^3 ^to 3.6 × 10^3 ^CFU/ml. The frequency of recombination was calculated from the puromycin- and G418-drug-resistant colony titers (Table 1). For the sequential transfection experiment the recombination frequencies ranged from 1 to 1.3 %, with an average of 1.1 %, while recombination frequencies for the cotransfection experiment ranged from 3.3 to 4.5 %, with an average of 3.9 %.

**Table 1:** The comparative study of recombination frequencies for cotransfected and sequentially transfected retroviral vectors

Method of introduction	Clone	Viral titer (CFU/ml)		Recombination frequency* (%)
			
		Puromycin	G418	
Sequential Transfection:				
pDΔpbsSVpuro + pDHEneo	ST1-1	5.1 × 10^1^	5.0 × 10^3^	1.0
	ST2-1	4.2 × 10^2^	4.2 × 10^4^	1.0
	ST2-2	8.0 × 10^2^	6.3 × 10^4^	1.3
	Mean ± SE			1.1 ± 0.1
Cotransfection:				
pDΔpbsSVpuro + pDHEneo	CT1	3.6 × 10^3^	1.1 × 10^5^	3.3
	CT2	1.4 × 10^3^	3.1 × 10^4^	4.5
	CT3	2.0 × 10^3^	5.5 × 10^4^	3.6
	CT4	3.1 × 10^3^	7.4 × 10^4^	4.2
	Mean ± SE			3.9 ± 0.3
pDΔpSVpuro + pDHEneo	CR1	2.5 × 10^1^	1.0 × 10^5^	0.03
	CR2	2.5 × 10^1^	4.8 × 10^4^	0.05
	CR3	0.9 × 10^1^	2.9 × 10^4^	0.03
	Mean ± SE			0.04 ± 0.01

*The frequency of recombination was calculated as the ratio of puromycin- to G418-drug-resistant colony titer.

The restriction enzyme marker differences in the leader regions of vectors provided a means to analyze the nature of recombinants in NIH 3T3 cells examined by PCR assay. Cellular DNA was analyzed from eight Puro^r ^NIH 3T3 cell clones obtained after infection with viruses produced by ST2-1 helper cell clone and eight cell clones obtained after infection with viruses produced by CT1 helper cell clone. This assay showed that all analyzed proviruses were recombinants between parental viruses, three of which were generated by template-switching in the 300 nt DLS region, and thirteen which were generated by template-switching in the 1038 nt region of 3' DLS (data not shown).

These experiments demonstrated that the frequency of recombination between vectors localized in the same chromosomal site was about four-fold higher than that of vectors with different chromosomal locations. These data suggest that there might be a preference for the formation of diploid retroviral genome from RNA molecules that are transcribed on the same DNA template. On the other hand, it could not be completely excluded that the high frequency of recombination for retroviral vectors in the cotransfection experiments occurred during or after transfection procedure.

### The use of retroviral vector with the inactivated promoter

To study the possibility of recombination between cotransfected vectors during or after transfection, we used the defective vector in which the 5' LTR promoter was deleted. This vector, pDΔpSVpuro, is almost completely homologous to pDΔpbsSVpuro with the exception of 194 bp in the U3 region (Figure 1). The efficiency of recombination during cotransfection for pDΔpSVpuro and pDHEneo was expected to be similar to that of pDΔpbsSVpuro and pDHEneo. However, the restoration of pDΔpSVpuro during reverse transcription will be limited by the basal level of cellular transcription since this vector is transcriptionally defective. Thus, the use of vector with the inactivated promoter could distinguish between recombination at the level of DNA and RNA in our experimental system.

The introduction of viral vectors into the packaging cell line, GP+envAM12, allowed selection and propagation of individual cellular clones under conditions similar to those in the previous experiments. The resulting viral titers are shown in Table 1. For three helper cell clones generated after the cotransfection with pDΔpSVpuro and pDHEneo the G418 titers varied from 2.9 × 10^4 ^to 1.0 × 10^5 ^CFU/ml, with an average 5.9 × 10^4 ^CFU/ml, and the puro titers varied from 0.9 × 10^1 ^to 2.5 × 10^1 ^CFU/ml, with an average 2.0 × 10^1 ^CFU/ml. The frequency of recombination for these vectors was 0.04 %. Thus, these results clearly demonstrated that recombination during cotransfection in our experimental system was a rare event and the majority of recombinations between cotransfected vectors occurred during the reverse transcription.

## Discussion

In the present work we have examined whether there was a preference in the formation of homodiploid genomes when two genetically different retroviral vectors were located in the different regions of the host genome. Since plasmid DNA transfected into eucaryotic cells is usually tandemly integrated [16-19], we have compared the frequencies of recombination for two Mo-MuLV-based retroviral vectors introduced into the helper cell line by either cotransfection or sequential transfection. Our results showed that cotransfection yielded about four-fold higher frequency of recombination comparing to sequential transfection, indicating that diploid retroviral genome is mainly formed from RNA molecules transcribed on the same DNA template. To exclude the possibility that recombination between vectors occurred during the cotransfection or/and the integration of plasmid DNA into the helper cell genome, we used a retroviral vector with the deletion of promoter-enhancer sequences as a partner for recombination. The 100-fold lower frequency of recombination for transcriptionally deficient vector, compared to that of the identical retroviral vector with the intact promoter, indicated that recombination during cotransfection was a rare event relative to recombination during reverse transcription.

Recent studies using the Moloney murine leukemia virus and the Spleen necrosis virus based vectors demonstrated that the recombination rate did not increase linearly with the increasing of marker distance and the multiple recombination events were observed much more often than could be expected from the frequency of recombination [10,15,21,22]. From these data it was postulated that the rate of retroviral recombination is restricted by the size of the recombining subpopulation [10,15,21]. On the other hand, the rate of recombination obtained for HIV-1 was about two to three crossovers per genome per replication cycle [11,12]. High rate of HIV-1 recombination was also observed in the experimental system where target sequences and experimental conditions for recombination were the same as in Mo-MuLV- and SNV-based studies [23]. While the rates of intermolecular recombination for HIV-1 and gammaretrovoruses were different, their intramolecular template switching frequencies were similar [14,24].

The preferential formation of homodimers in the mixed retroviral infection can explain the existence of the recombining subpopulation found for avian and murine retroviruses because, in this case, the amount of heterodiploid virions will be less than expected from the randomly distributed genomic RNA. Our demonstration of about 4-fold differences in the frequencies of recombination for the cotransfected and sequentially transfected retroviral vectors seems to agree with the data showing that the maximal recombination rate for Mo-MuLV was 20 % per genome per replication cycle [10,22]. These data also indicate that the difference in the recombination frequencies for gammaretroviruses and HIV-1 could mainly be associated with the ability of these viruses to copackage two different genomic RNAs.

The possible mechanism explaining the preferential formation of homodimers, as suggested earlier [15], may be a local transport of RNA transcribed in the same locus of chromosome from the nucleus to their destination in the cellular cytoplasm. In the cytoplasm, RNA could be quickly bound by viral proteins before two different pools of RNA molecules transcribed in different chromosomal sites will be equally distributed. The gammaretroviruses and HIV-1 could differ in the properties of their RNA transport and distribution in the cellular cytoplasm. For example, HIV-1 encodes the virus-specific protein Rev which selectively transports the unspliced viral RNAs from the nucleus to cytoplasm [25]. Moreover, unspliced HIV-1 RNAs form a general cytoplasmic pool of molecules which can further participate in the translation of viral proteins and/or be packaged in the virions [26]. It was recently shown that translation of HIV-1 viral RNAs could precede their packaging [27]. In this case, the translation of genomic RNAs can provide more time for reassortment of two different viral RNAs. As an alternative, it can be suggested that the dimerization of genomic RNAs of gammaretroviruses occurs immediately after transcription in the cell nucleus and heterodimerization involves only minor populations of RNA molecules left in a monomeric form and/or unstable homodimers.

The diploidy of retroviral genome supposes that two molecules of RNA could be necessary for replication of virus. However, it is also possible that diploidy is important for recombination and evolution of virus since retroviruses do not have a pool of replicative intermediates that can undergo recombination [5]. The preferential copackaging of genetically identical retroviral RNAs further argues in favour of the hypothesis that both RNA molecules are required in each round of retroviral replication. This assumption is also in agreement with the results of previous studies showing the utilization of both HIV-1 RNAs during reverse transcription [11,12]. It can be suggested that two genomic molecules of RNA are necessary to repair frequent breaks in RNAs [28] or the synthesis of provirus requires involvement of cis-acting elements present in both RNA molecules.

Upon completion of our manuscript, an article was published concluding that dimerization of Mo-MuLV genomic RNAs is carried out by nonrandom chance [35]. There are several differences in these two studies. In the cited report, the RNA dimers were examined in the viruses that were generated by transiently cotransfecting two vectors or were produced by cell clones containing retroviral vectors integrated in different chromosomal sites. A model of nonrandom dimerization has been proposed, where Mo-MuLV genomic RNAs may undergo dimerization cotranscriptionally. In our study, the frequencies of recombination were directly compared for cell clones where retroviral vectors were integrated in the same or different chromosomal sites. While retroviral vectors integrated in the same chromosomal site were expressed as independent transcriptional units, the efficiency the heterodimer formation was increased about four-fold compared to that of retroviral vectors with different chromosomal locations. This argues that dimerization of Mo-MuLV genomic RNAs during cotranscription is not the main reason for the preferential formation of homodiploid genomes in Mo-MuLV. In spite of substantial differences in the methods, the estimations of the efficiency of homodimer formation were similar in both studies. The experimental system presented in our report could be used to study cellular and viral factors that are responsible for the preferential copackaging of genetically identical retroviral RNAs.

## Conclusions

The results of this study provide evidence that the Mo-MuLV genome is mainly formed from RNA molecules synthesized on the same DNA-provirus. This property of Mo-MuLV may explain why only small subpopulations of gammaretroviruses produce recombinants. In this context, the differences in the frequencies of recombination between HIV-1 and Mo-MuLV may reflect differences in the ability of these viruses to randomly copackage genetically distinct RNAs. The preferential formation of homodiploid genomes in Mo-MuLV also implies that both molecules of RNA might be required for replication of the retroviral genome.

## Methods

### Plasmid constructions

pMOV9 containing the complete copy of Mo-MuLV provirus and retroviral vectors pDneo and pDSVpuro have been described earlier and were used as the progenitor for all the constructions [29,30]. pDneo and pDSVpuro contain upstream long terminal repeat (LTR) and ψ^+ ^region before position 1560 of Mo-MuLV sequences [20], neomycin phosphotransferase gene or puromycin N-acetyltransferase gene under control of Simian virus 40 (SV40) early promoter region, and the Mo-MuLV sequences from position 7674 including downstream long terminal repeat. The nucleotides are numbered for the Mo-MuLV sequences starting from the beginning of R region [20]. To generate pDΔpbsSVpuro, we first constructed pLTRΔpbs which contains the LTR and the leader region before position 564 of pMOV9 with the deletion of PBS region. For this purpose we used the PCR to amplify two overlapping fragments after joining of which the PBS region was substituted with the EcoRI site. The first PCR fragment was generated with the primers: U3 SalI 5'-CGCGTCGACAGAAAAAGGGGGGAA-3' (sense, positions 7803–7821) and Rir EcoRI 5'-GCGCGAATTCAATGAAAGACCCCCG-3' (antisense, positions 130–144); the second PCR fragment was generated with the primers: 3'PBS EcoRI 5'-GCGCGAATTCCGGGAGACCCCTGCC-3' (sense, positions 164–178) and L2 5'-GACAAATACAGAAAC-3' (antisense, positions 599–613). PCR fragments were digested with EcoRI, ligated, and further digested with SalI and PstI, and cloned into pBluescript KSII^+ ^(Stratagene). The amplified region of pLTRΔpbs was analyzed by sequencing. The resulting construct, pDΔpbsSVpuro, was generated by exchanging the KpnI-PstI (nucleotide positions 32 to 564) fragment of pDSVpuro with the corresponding fragment of pLTRΔpbs.

pDHEneo is identical to pDneo except the point mutations in the sequences flanking the DLS region. These mutations converted the Mo-MuLV sequences in this region into new restriction sites for HindIII and EcoRI. A description of the cloning steps performed to generate this vector is available upon request.

To produce pDΔpSVpuro, the enhancer-promoter sequences of U3 region in pDΔpbsSVpuro were deleted. For this purpose the 3.4 kb SacI-BamHI fragment containing 36 bp of 5' U3 region starting from SacI site and including all other vector sequences of pDΔpbsSVpuro was inserted into the SacI and BamHI sites of pTZ18 plasmid.

All DNA manipulations were performed by standard procedures [31].

### Analysis of integrated plasmid DNA

Genomic DNA purification and hybridization were performed by standard molecular techniques [31]. DNA prepared from double-drug-resistant cell clones was used as a substrate for PCR. Integrated plasmid DNA was amplified using a 3' neo-specific sense primer T1 (5'-AGTGCAAATCCGTCGGCAT-3') and an antisense primer T2 (5'-GAGGCGGCCTCGGCCTC-3') within the SV40 early promoter. The sequences of neo gene in proviral DNA were PCR amplified using primers CND (5'-CACGCTGCCGCAAGCACTCA-3') and CNR (5'-TGGGTGGTGAGCAGCTCGCC-3'). PCR was performed in 10 mM Tris (pH 8.3), 50 mM KCl, 2 mMMgCl_2_, 200 μM each dNTP, 1 % DMSO, 100 nM primers for 20 cycles (94°C 1 min, 50°C 1 min, 72°C 8 min). The products were separated on 0.8 % agarose gel, transferred onto nylon membrane (Hybond-N, Amersham), and hybridized with neo-specific probe (150 bp SalI-ClaI fragment of pDHEneo). Probes were generated by the random-primer method with [α^32^P] dATP [32].

### RT-PCR analysis

Virion RNA was purified from filtered culture medium from transfected cells and used in RT-PCR assays [31]. Briefly, RNA samples were reverse-transcribed in a 20-μl reaction with Superscript II (Life Technologies), using an antisense gag-specific primer (L2) beginning at nt 613 (5'-CAAAGACATAAACAG-3'). A third of the resultant cDNA was subjected to PCR (94°C for 1 min, 50°C for 1 min, 72°C for 1 min, for 30 cycles) with AmpliTaq DNA polymerase (Perkin-Elmer), using the same primer that was used in the RT reaction and paired with a sense R-specific primer (L1) beginning at nt 1 (5'-GCGCCAGTCCTCCGA-3'). PCR products were digested with 10 units of EcoRI (Fermentas) according to the manufacturer's recommendations and analyzed by 2 % agarose gel. A GelDoc™ EQ system (Biorad) with SigmaGel v.1.0 software (Jandel Scientific) was used to quantitate the ethidium bromide fluorescence intensity of each band.

### Cells, DNA transfection, and virus propagation

NIH3T3 (murine cell line) and GP+envAM12 (amphotropic 3T3-based packaging cell line with MLV Gag + Pol and Env genes) [33] were grown in Dulbecco's modified Eagle's medium supplemented with 10 % fetal calf serum.

The cell clones producing transfected vectors were established by transfecting GP+envAM12 cells with vector plasmids using the dimethyl sulfoxide-polybrene method [34]. Puromycin-resistant cells were selected in 2.5 or 1.5 μg/ml puromycin (Sigma) for GPenv-AM12 or NIH3T3-derived cells, respectively. Geneticin selection was performed at 800 μg/ml (GP+envAM12) or 600 μg/ml (NIH3T3) of G418 (Gibco).

Viral infection was performed immediately after harvesting the virus. The supernatants were harvested from 90 % confluent stable producer cell clones after 16 hour intervals and filtered through the 0.45 μm filters. Infections were performed in the presence of 8 μg/ml polybrene (Sigma) for two hours at 37°C. Puromycin- and G418-resistant cfu titers were determined using the infection of NIH3T3 cells by end-point dilution.

## Competing interests

The authors declare that they have no competing interests.

## Authors' contributions

SAK carried out most experiments and made substantial contributions to conception and design. AIK and ABO carried out analysis of integrated plasmid DNA by hybridization and participated in the works with cell cultures. IKF conceived of the study, participated in the design and coordination, and drafted the manuscript. All authors read and approved the final manuscript.
